# Neuroscientific Model of Motivational Process

**DOI:** 10.3389/fpsyg.2013.00098

**Published:** 2013-03-04

**Authors:** Sung-il Kim

**Affiliations:** ^1^Department of Education, Brain and Motivation Research Institute, Korea UniversitySeoul, South Korea

**Keywords:** motivation, neuroeducation, educational neuroscience, reward, value, goal, decision-making, self-regulation

## Abstract

Considering the neuroscientific findings on reward, learning, value, decision-making, and cognitive control, motivation can be parsed into three sub processes, a process of generating motivation, a process of maintaining motivation, and a process of regulating motivation. I propose a tentative neuroscientific model of motivational processes which consists of three distinct but continuous sub processes, namely reward-driven approach, value-based decision-making, and goal-directed control. Reward-driven approach is the process in which motivation is generated by reward anticipation and selective approach behaviors toward reward. This process recruits the ventral striatum (reward area) in which basic stimulus-action association is formed, and is classified as an automatic motivation to which relatively less attention is assigned. By contrast, value-based decision-making is the process of evaluating various outcomes of actions, learning through positive prediction error, and calculating the value continuously. The striatum and the orbitofrontal cortex (valuation area) play crucial roles in sustaining motivation. Lastly, the goal-directed control is the process of regulating motivation through cognitive control to achieve goals. This consciously controlled motivation is associated with higher-level cognitive functions such as planning, retaining the goal, monitoring the performance, and regulating action. The anterior cingulate cortex (attention area) and the dorsolateral prefrontal cortex (cognitive control area) are the main neural circuits related to regulation of motivation. These three sub processes interact with each other by sending reward prediction error signals through dopaminergic pathway from the striatum and to the prefrontal cortex. The neuroscientific model of motivational process suggests several educational implications with regard to the generation, maintenance, and regulation of motivation to learn in the learning environment.

## Introduction

Since early theories of biological motives such as hunger, thirst, and sex have been proposed, research on diverse aspects of human motivation has been conducted to extend its conceptual boundaries and understand the dynamics of motivation. As a result, we have major psychological theories on motivation including reinforcement learning theory, need theory, attribution theory, self-efficacy theory, self-determination theory, expectancy-value theory, achievement goal theory, interest theory, and self-regulation theory. There is no doubt that these theories have contributed in deepening our understanding of complex human motivation, but it’s time for a new approach to overcome the fundamental limitation of traditional theories.

Existing theories on motivation bear three limitations. First is the vagueness of the concept of motivation. It is practically impossible to draw a clear line between motivation and other concepts such as drive, need, intention, desire, goal, value, and volition. Due to this conceptual vagueness, it is difficult to come to a consensus on whether motivation refers to an psychological state or process, let alone the definition. Various constructs in different theories of motivation are overlapping and often create confusion. For instance, the vague conceptual distinctions between intrinsic motivation and interest, self-efficacy and perceived competence, value and reward, self-regulation and volition hinder effective communication and constructive arguments on the identical phenomenon of motivation.

Second limitation is the absence of comprehensive theory on motivation. Although a number of theories on motivation have been proposed, each one deals with only a specific fraction and it lacks profound understanding of motivational process as a whole. The measurement of motivation is the third limitation. Action selection, frequency and persistence of the action, and the degree of time and effort put into sustaining the action are direct indicators of motivation. Although these measurements can be obtained objectively through a long-term observation, due to practical limitations, they are mostly conducted in the form of self-report surveys on psychological constructs that are highly correlated with behavioral measurement. However, as motivation is largely implicit and dynamic, the measurement relying on self-report is very much restricted to consciously accessible aspect of motivation.

Due to these limitations, extensive research on motivation so far is yet to provide practical implementation into schools or workplaces. For effective motivational interventions, we need to set a clear conceptual definition of motivation and come up with a comprehensive conceptual framework that integrates diverse perspectives. Measuring the brain activation pattern using neuroimaging techniques can be a complementary way of overcoming above-mentioned limitations. By detecting the changes in the brain during task performance, it became possible to understand the dynamic yet implicit nature of motivation.

**Figure 1:**
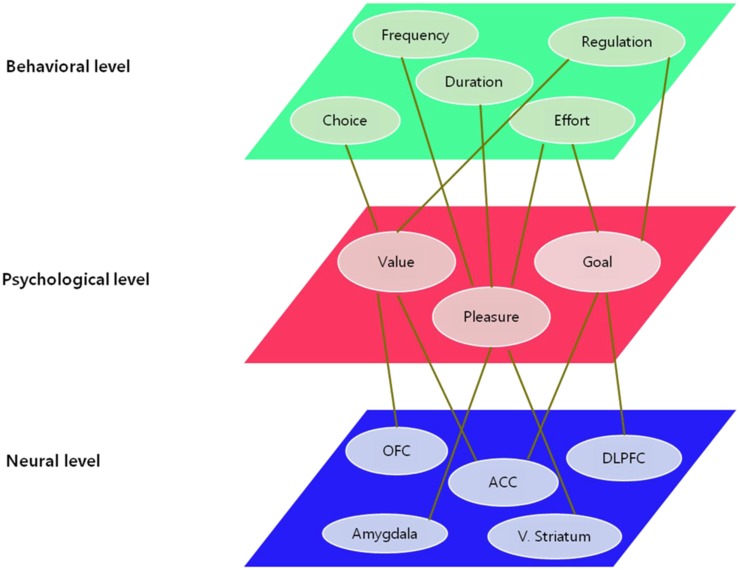
**Levels of explanation and units of analysis on motivation**.

I propose a tentative model of motivational processes which focuses on the various stages of being motivated, based on converging evidence in cognitive neuroscience, affective neuroscience, social neuroscience, developmental neuroscience, and neuroeconomics. In order to fully understand a phenomenon, it can take more than a single unit of analysis which determines the level of explanation. The more converging evidence from diverse levels of explanation are provided, the more precise the understanding of the phenomenon it becomes. The same goes for motivation. Diverse units of analysis and levels of explanation coexist; from microscopic molecular perspective to macroscopic socio-cultural perspective. As shown in Figure 1, the unit of analysis draws the distinction among different levels of explanation: neuronal level, psychological level, and behavioral level.

The neuronal level of explanation describes the motivation-related phenomena as functions of the ventral striatum involved in reinforcement learning, the orbitofrontal cortex (OFC) region linked to value judgment and decision-making, and the anterior cingulate cortex (ACC) and the dorsolateral prefrontal cortex (DLPFC) regions associated with executive function and cognitive control. On the other hand, the units of analysis in the behavioral level of explanation refer to the frequency and persistency of the action, the degree of effort and engagement, selection of approach and avoidance behavior, regulatory behavior, and so on. The psychological level of explanation has mainly considered constructs such as reward, expectation, value, goal, attribution, competence, interest, and self-regulation as the primary units of analysis. However, these are somewhat ambiguous and overlapping psychological constructs which may not correspond to units of analysis in the neuronal level. With the rapid advance in the field of neuroscience, the validity and conceptual clarity of these psychological constructs can be complemented by neuroscientific evidence, and thereby the fundamental reconceptualization on the psychological level has become active (e.g., Rangel et al., 2008; Heatherton, 2011).

**Figure 2:**
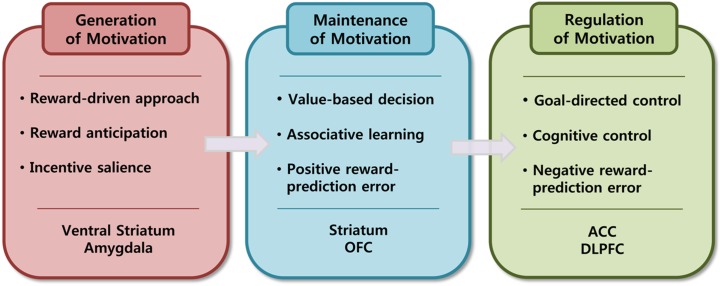
**Three sub proceses of motivational process**.

In this paper, I focused on pleasure, value, and goal as principal units of analysis on psychological level because their underlying neural mechanisms have been heavily investigated and relatively well identified. I also try to propose a neuroscientific model of motivational processes in which motivation is regarded as a dynamic process and is understood as a series of detailed sub processes of generation, maintenance, and regulation of motivation. I will explain the generation of motivation in terms of the reward-driven approach process, the maintenance of motivation in terms of the value-based decision-making process, and the regulation of motivation in terms of goal-directed control process (See Figure 2).

## Generation of Motivation: Reward-Driven Approach Process

### Role of reward

One of the most powerful variables influencing motivation is reward, irrespective of reward type (physical or social reward). The main function of reward is to induce positive emotions, make the organism approach, increase the frequency of the target behavior, and hence prevent extinction (Schultz, 2004). As a result, the organism looks for predictive reward signals, acquires the stimulus-reward association, encodes the value of reward, and decides on approach or avoidance behavior to acquire the sustainable reward. However, the reward mechanism is not simple when the associative learning via reward, reward-based decision-making, and behavioral control to obtain future reward are taken into account. The reward processing consists of a sequence of sub processes such as anticipating the reward, associating reward with behavior, planning to obtain the reward, encoding the value of reward, and updating the relative value of reward. Thus, diverse brain regions are recruited during reward processing.

**Figure 3:**
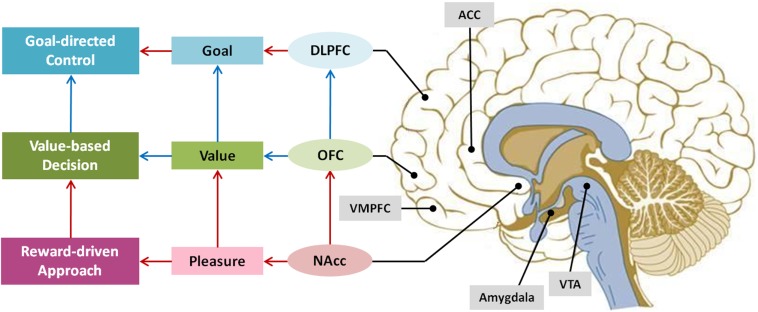
**Key brain regions related to motivational process**.

The primary brain regions associated with reward is the dopamine pathway widely known as reward pathway. Dopamine is a neurotransmitter that is produced in the ventral tegmental area (VTA), passes through the globus pallidus and released into the nucleus accumbens (NAcc) located in the striatum (See Figure 3). This pathway is divided into mesolimbic dopamine system and mesocortical dopamine system. Mesolimbic dopamine system is where VTA neurons are connected to the NAcc, the septum, the amygdala, and the hippocampus; and mesocortical dopamine system is the linkage between the medial prefrontal cortex (MPFC), the ACC, and the perirhinal cortex. The mesolimbic dopamine system is responsible for reward anticipation and learning, whereas the mesocortical dopamine system involves in encoding the relative value of the reward and goal-directed behavior.

The OFC, the amygdala, and the NAcc are the brain regions that are consistently reported to be involved in reward processing (e.g., Haber and Knutson, 2010). The DLPFC, the MPFC, and the ACC are also reported to be relevant to reward processing, but the primary functions of these areas are more to do with the executive function in achieving the goal (Miller and Cohen, 2001; Walter et al., 2005). The OFC-amygdala-NAcc system responds not only to primary rewards such as food or sexual excitement but to secondary rewards like money and social rewards including verbal praise or cooperation (Rilling et al., 2002; Kringelbach et al., 2003; Izuma et al., 2008). In particular, the NAcc known as the pleasure center is activated when a variety of rewards are anticipated or received. For instance, the NAcc is activated when people were presented with favorite stimulus, engaged in favored activity, smoking, hearing jokes, and even when feeling love (Aharon et al., 2001; Mobbs et al., 2003; Aron et al., 2005). In contrast, several studies show that amygdala, which is known to respond to conditions associated with fear and negative stimulus, is intensity-sensitive not valence-sensitive (Anderson et al., 2003; Small et al., 2003).

In case of primary reward, which is essential for survival that all the species are automatically programmed to approach it, the ventral striatum including the NAcc forms an association of behavior-outcome. However, in case of conditioned secondary reward, the OFC encodes and represents the associative value of reward, and updates the value for future decision-making process. The OFC is the critical brain region for value judgment (Grabenhorst and Rolls, 2011). In particular, the medial OFC is reward-sensitive, whereas the lateral OFC is punishment-sensitive (O’Doherty et al., 2003). Tremblay and Schultz (1999) have discovered that the OFC does not respond to the absolute value of the reward but it calculates the relative value of the reward and respond only to the ones with higher preference. This finding is in line with Premack’s principle which states that reward is highly subjective and relative and suggests that there is no such thing as universal reward which goes beyond the individual characteristics and specificity of the situation.

Social neuroscience research has discovered that both social and physical rewards/punishments activate the same area of the brain (Lieberman and Eisenberger, 2009). In other words, social rewards such as reputation, fairness, cooperation, and altruistic behavior activate the reward-related network that is activated when experiencing physical pleasure. Social aversive stimuli such as social exclusion, unfair treatment, and social comparison activate the pain-related brain regions. These results suggest that social reinforcement and punishment are as powerful and effective as physical reward and pain. It is important to conduct neuroeducational studies to investigate whether social stimuli such as compliment, encouragement, support, empathy, cooperation, fairness, and altruistic behavior activate the reward pathways of children and adolescents, and to design a learning environment that allows sustainable activation of reward pathways. Based on these findings on how bullying, normative grading, competition, discrimination, punishment, and penalty systems at schools are affecting the students’ brain development, we can suggest possible solutions to minimize these demotivating features of the learning environment. Kim et al. (2010), for example, conducted a study where learners were given feedback on their performance in the form of absolute assessment and relative assessment, and their brain activation patterns were compared during feedback. The result showed that when relative assessment was given to low competence learners, the amygdala, a brain region associated with negative emotions, was activated even if the feedback valence was not negative. This suggests that relative assessment should be used with caution, especially for learners with low competence, because it produces negative affect regardless of their actual performance.

One important issue in relation to reward is the distinction between intrinsic and extrinsic motivation. If intrinsic and extrinsic motivation are different constructs, is it possible to biologically distinguish them and find dissociable neural mechanisms underlying each type of motivation? No neuroscientific evidence has yet been found to support this claim. Moreover, it is quite common that extrinsic and intrinsic value for a specific behavior coexists. Since each reward has specific value which is subjectively computed, whether the source of the value is internal or external may not carry any significant meaning in value computation process.

People are motivated to behave to obtain desirable outcomes, and also to avoid negative consequences. But under rapidly changing circumstances where the consequence of the action is uncertain, a decision has to be made whether to stick to one’s current strategy or to look for new alternatives. The trade-off between these two options is known as exploration-exploitation dilemma in reinforcement learning (Sutton and Barto, 1998). In exploratory learning where new alternatives are sought, both the frontopolar cortex and the intraparietal sulcus responsible for value judgment and inference are activated (Daw et al., 2006). On the other hand, in exploitative learning where habitual decision-making occurs based on prior experience, the striatum and the MPFC are activated. That is, the striatum and the amygdala are responsible for approach and avoidance behavior respectively which are modulated by the prefrontal cortex. According to the studies on brain development, the NAcc which is sensitive to reward shows rapid development in adolescence, whereas the amygdala that plays a key role in avoidance of danger shows rather slow development, and the prefrontal cortex responsible for controlling of action shows the slowest development (Ernst et al., 2005; Casey et al., 2008). Therefore, adolescents are likely to demonstrate behavioral tendencies that are more close to exploitation than exploration. This imbalance between limbic system and prefrontal cortex in adolescent brain development provides an understanding of impulsive, sensation-seeking, and risk-taking behaviors of teenagers.

In the study on the sensitivity to reward, adolescents showed greater activation in the NAcc while receiving the reward than adults (Galvan et al., 2006; Ernst and Frudge, 2009), but the opposite pattern was true while anticipating the reward (Bjork et al., 2004). Additionally, in the adult brain, the OFC was activated when the expected reward was not given (Van Leijenhorst et al., 2010). This suggests that existing value system of the adult is probably being updated to pursue successive rewards when the expected reward is not given. Adolescents are known to be more sensitive to rewards or positive feedback but less to punishments or negative feedback than adults (Bjork et al., 2004). The comparison of brain activation by age groups showed the differential activation pattern in the DLPFC. For the children aged 8–9, it was activated when positive feedback was given, whereas for children aged 11–13 it was activated in response to both positive and negative feedbacks. For adults aged 18–25, it was activated only when negative feedback was given (Duijvenvoorde et al., 2008). This developmental difference suggests that negative feedback for young children might not be effective due to the slow development of the prefrontal cortex.

### Distinction between liking and wanting

The intrinsically motivated activity does not necessarily accompany hedonic enjoyment. For example, although a soccer player may have a strong intrinsic motivation to play soccer, sometimes he may not feel pleasure during physical training or soccer practice. Spontaneous goal-directed actions are inherently motivated, but instrumental actions to achieve a goal can be temporarily unpleasant. The new contention that pleasure and enjoyment are not sufficient conditions for intrinsic motivation has been gaining recognition. According to Berridge (2007), positive emotions and intrinsic motivation do not coincide all the time and they are operated by different physiological mechanisms. In other words, the persistent approach behavior toward a specific stimulus does not necessarily mean that the stimulus is favored. Berridge and Robinson (2003) suggested that reward has two values; hedonic value reflecting the degree of liking and incentive value reflecting the degree of wanting. Whereas “liking” is a passive state in which the quality of the stimulus is evaluated after being processed, “wanting” is an active state where stimulus is pursued before being processed. Wanting is not a state of desire like drive or craving, but a process that a specific stimulus embodies attractive value on the sensational or cognitive level. In order to distinguish “wanting” from its commonsensical meaning, it is often referred to as incentive salience.

Olds and Milner (1954) conducted a seminal experiment on the function of NAcc known as the pleasure center using a Skinner box. An electrode connected to a lever was implanted in the NAcc of a rat so that the NAcc was stimulated whenever the rat pressed the lever. They observed that the rat continuously pressed the lever without eating to stimulate the NAcc until it became totally exhausted. As it was impossible to conduct a self-report to verify whether the rat actually liked the electrical stimulation, a new method of measuring the emotion was needed. One widely used method of measuring pleasure or pain in infant or animal studies is to analyze the specific pattern of facial expressions in response to various kinds of taste, which is a universal indicator of emotions across species (Berridge, 2000). An animal study demonstrated that the NAcc of a rat was activated by drugs such as cocaine but its facial affective expression in response to the drugs was a disliking reaction (Berridge and Valenstein, 1991). This may in part explain why drug addicts constantly want the drug but they do not actually like it. Berridge (2003) also revealed that the brain regions responsible for liking and wanting are anatomically separated within the NAcc. These findings support the notion that persistent action to obtain specific stimulus is not necessarily pleasure-seeking one and wanting can occur without liking. By contrast, liking without wanting can be found in a study where the release of dopamine is suppressed by lesions or dopamine antagonists. In this case, no wanting behavior toward reward was shown, but there was no decrease in the degree of liking for the reward (Berridge and Robinson, 2003). Hence, it can be concluded that dopamine plays a key role in wanting the stimulus and increasing incentive salience, but it does not affect the liking for the stimulus.

Theories of intrinsic motivation and interest posit that people are intrinsically motivated to persistently engage in the activity when they experience pleasure and enjoyment (Csikszentmihalyi, 2000; Ryan and Deci, 2000; Hidi and Renninger, 2006). However, if “liking” and “wanting” are dissociated, motivation is not generated by feeling pleasure or liking the activity without wanting or incentive salience. This means that a state of liking for a specific object or activity cannot be understood as a motivational state and that liking is not a prerequisite for generating motivation. From this perspective, liking refers to an emotional state whereas wanting has more to do with motivation and decision utility (Berridge and Aldridge, 2008). There is a need for careful reconsideration for the argument in which the school activity should be enjoyable to generate motivation because pleasure and enjoyment may not automatically lead to motivation. Hence, the transition from liking to wanting and the relationship between motivation and emotion remain an important issue. Moreover, applying the aversive conditioning to behavior modification, which makes undesirable behavior less attractive, has to be cautiously examined because the assumption that people like their habits may be wrong.

Another new hypothesis about the function of NAcc is that dopamine plays a role in effort-related behaviors. The traditional hypothesis that dopamine is associated with the reward function has recently been criticized. These criticisms are based on the finding that the NAcc dopaminergic system is not involved in the pleasure relevant to the positive reinforcement, but is responsible for behavioral activation and effort-related functions (Salamone et al., 2007). An animal study on the effect of dopamine dosages demonstrated that dopamine depletion caused longer response time and severe deterioration in high-effort task performance. Also, rats with insufficient dopamine are prone to choose tasks requiring less effort over tasks requiring much effort (Salamone et al., 2005). According to these studies, the NAcc dopaminergic system may modulate the effort regulation rather than reward-related function. The brain regions associated with effort-based decision-making include an extensive circuit from the thalamus, the amygdala, and the ACC to the prefrontal cortex, but the NAcc is the key area to interact with these areas.

## Maintenance of Motivation: Value-Based Decision Process

### Reward prediction error and learning

No motivation is sustained without learning and memory. Approach-avoidance behaviors are learned and goal-directed behaviors depend on working memory. Because remembering actions that result in positive or negative outcome is beneficial in adaptation, stimulus-action-outcome association is learned and actions become habitual and automatic. Dopamine is known to be mainly associated with reward and pleasure, but it is a neurotransmitter that also plays an important role in motor performance, conditioning, learning, and memory (Wise, 2004). Insufficient dopamine causes stiffness and paralysis seen with patients with Parkinson’s disease, whereas excessive dopamine may result in behavioral disorders such as schizophrenia, impulse control disorder, ADHD, and addiction. After being injected with dopamine as treatment for Parkinson’s disease, the patients showed a marked increase in the compulsive behaviors such as excessive gambling or eating disorders (Dagher and Robbins, 2009). Functional disorders associated with excessive dopamine are not being able to control the dominant motor response, focusing more on gains than losses, making hasty and risky decisions, favoring small but immediate reward and so on.

According to reinforcement learning theory, the magnitude of learning depends on the dopamine release (Montague et al., 1996). Both positive reinforcement accompanying appetitive stimuli and negative reinforcement removing aversive stimuli increase dopamine release, which in turn increases the frequency of the target behavior and leads to associative learning between stimuli and behavior. Through repeated association with neutral stimuli (environmental stimulus or psychological state), the powerful association of stimulus-action-outcome is learned. The initial reward for a chosen action is most likely unpredictable, so the effect of the reward is maximized. This difference between the expected and the actual reward is referred to as reward prediction error (RPE), which is encoded by dopaminergic neurons. The larger the RPE is, the more dopamine is released. In a study conducted by Schultz et al. (1997), they examined the response of a single dopamine neuron. At an early stage of learning when the chimpanzee did not expect a reward, dopamine neuron was activated while receiving reward. However, when a reward was always anticipated due to repetition, the dopamine neuron was activated only when cues for the reward were given, and it was not actually activated while receiving the reward. On the contrary, when the expected reward was not given, dopamine neuron was suppressed. This shows clearly that it takes only anticipation for the reward, through various conditioned stimuli associated with reward or punishment, not the reward itself, to boost the dopamine release and hence to generate the target behavior. This is a very beneficial way of learning from the perspective of adaptation.

There are two types of RPE: positive and negative RPE (Schultz, 2006). Positive RPE is generated when the outcome is better-than-expected or unexpected rewards are given, whereas negative RPE is generated when the outcome is worse-than-expected or expected rewards are omitted. The larger the positive RPE, the bigger the surprise, hence maximum learning occurs. Repeated use of rewards, however, increase the expectation of reward at all times reducing positive RPE, so it reaches an asymptote (close to zero) without learning gains. For this reason, the typical learning curves are negatively accelerated, indicating that rapid growth occurs at early stages of learning but this increment gets smaller on later stages.

To maintain students’ motivation for target behavior, a certain amount of dopamine should be released during or after the pursuit of the target behavior. The dopamine can be released by the positive RPE whenever the unexpected positive outcomes are given. At this point, a specific action is sustained as long as the outcome of a habitual action is satisfactory. In order to maximize the learning, it is essential to provide relatively new reinforcements to increase positive RPE. It is highly consistent with interest theory which posits the importance of providing the unexpected stimuli which can be easily resolved later, such as novel or surprising stimuli with cognitive gap or conflict (Berlyne, 1974; Kim, 1999). However, one of the dilemmas among educators is that any kind of learning requires practice through repetition which usually undermines the motivation. Thus, if the instructors cannot help but repeat the learning material, then they should introduce a new learning activity or novel learning context in order to produce positive RPE.

The clear example of the motivation-learning link is addiction. Excluding serious malfunctions in controllability, an addicted behavior is not only the most powerfully motivated action but also the result of maximum learning. Once the cue-reward association is learned, the role of the cues to activate the dopamine system grows, but the role of reward itself gets smaller. That is because the brain has a strong tendency to reduce dopamine release when the reward is expected (Self, 2003). However, in the case of psychoactive substances such as alcohol or cocaine, dopamine is excessively released without the typical learning process. As a result, extreme memory or pathological learning is induced to recognize these substances as new and salient rewards (Hyman et al., 2006). This explains why it is difficult to break the drug addiction and why only a single exposure can lead to a relapse even after a long period of abstinence. Behavioral addictions including online game addiction which is common among adolescents, are also reported to show a similar pattern to drug addiction (Grant et al., 2006), but more systematic studies need to be conducted to reveal the precise mechanism.

### Outcome evaluation and action selection

Numerous behaviors in everyday life are determined by the choice from many other alternatives whether to continue or to stop a specific action. Action selection is a part of decision-making process based on value assessment. The higher the assessed value of the outcome from the selected action, the greater the possibility of choosing it later. Rangel et al. (2008) distinguished three different types of valuation systems which play an essential role in decision-making process; Pavlovian, habitual, and goal-directed system. Pavlovian system assesses values with regards to the salience of stimulus. The network of the amygdala, the NAcc, and the OFC is involved in this process. Habitual system evaluates the value of stimulus-response association following the reward. The dorsal striatum and the cortico-thalamic loops are the main brain regions for this system. Lastly, goal-directed system calculates the association of action-outcome and evaluates the reward assigned to other outcomes. The OFC and the DLPFC are responsible for this process. Let’s take studying as an example of value-based decision-making. Pavlovian system assesses the value of a specific school subject such as English, habitual system evaluates the action of studying English vocabulary every morning, and goal-directed system determines which subject to concentrate on during vacation.

To make an effective choice, it is required to judge the potential value of the action which reflects the probability of its desirable outcomes. This is referred to as expected utility in economics and psychology. The brain should calculate the expected value before the choice is actually made. In an experiment where the magnitude and probability of the reward were manipulated, the NAcc activation showed a positive correlation with the magnitude of expected reward and positive emotions, and the activity of the MPFC had a positive correlation with the probability of obtaining the reward (Knutson et al., 2005). This finding indicates that the subcortical structure responds mainly to physical property and emotional aspect of reward, whereas the prefrontal cortex is more involved in the higher order computational function associated with the probability of obtaining the reward. In case where unexpected outcome is resulted from a choice, recomputation or update of the value of the action is required. Hence, children and elderly with low prefrontal cortex function tend to find it difficult (Brand and Markowitch, 2010).

The OFC, a core brain region for value judgment and decision-making, is also known as a reward-related region, but its role in reward processing is not straightforward (Kringelbach, 2005). Animal studies have demonstrated that animals with OFC lesions were capable of normal reward processing. They were able to perform actions to obtain rewards, learn the associations between stimuli and new rewards, and distinguish rewards from no rewards (Izquierdo et al., 2004; Rudebeck et al., 2006). This indicates that the primary function of OFC is to integrate every aspect of information, calculate the value to expect the outcome of the choice, and represent it in working memory (Montague and Berns, 2002). According to the somatic marker hypothesis by Damasio (1996), somatic states related to various emotions, which were generated during the process of evaluating actions, influence the final decision. The function of OFC in this process is to encode somatic state associated with the environmental pattern and retrieve it to recalculate the value for future decision-making. A neuroimaging study demonstrated that the OFC responded not only to sensory stimuli inducing pleasant-unpleasant odor and sound, but to abstract reward and punishment such as making money and losing it (Rolls and Grabenhorst, 2008). The OFC is in close connection with adjacent prefrontal regions and constantly interacts with them to search for more effective decision-making. More specifically, the OFC calculates the value, whereas the DLPFC retains this information to plan actions for the reward and the MPFC evaluates the effort required to execute the plan (Wallis, 2007; Grabenhorst and Rolls, 2011).

The function of OFC becomes clear when we take a close look at the cases with OFC damage. The most famous case is Phineas Gage who was working as a railroad construction foreman when he was involved in an accident in which a metal rod was driven through his head. He survived the accident and displayed normal cognitive abilities but he exhibited inappropriate behaviors in social interactions such as erratic and impulsive behaviors. This case drew attention as the first case to demonstrate the possible relation between prefrontal cortex including the OFC and social skills or personalities. Patients with OFC damage are not cognitively impaired, but show severe defects in daily decision-making and tend to exhibit obsessive-compulsive disorder, drug or gambling addiction, and eating disorder (Camille et al., 2004). It is also known that patients find it difficult to control emotion and they do not usually experience regret (Camille et al., 2004). Feeling of regret occurs when an individual compares the outcome of current choice with possible alternatives, but patients with OFC damage are thought to have problems with this counterfactual thinking.

While conducting a gambling task, patients with OFC damage persist in high-risk (low probability of winning) choices to win large money at once and they ultimately lose all the money (Bechara et al., 1994). This impulsive behavior is closely related to the NAcc which is connected to the OFC. The OFC controls the immediate response of the NAcc. If the NAcc is not controlled due to the OFC malfunctions, it is difficult to suppress impulsive behaviors. The suppressive role of the OFC controlling the NAcc can be explained as a general function of the OFC, value representation and value computation. That is, the suppression of impulse is a result of Go/NoGo decision based on comprehensive value assessment. The OFC assesses the values on the expected outcomes of each action and signals them. When the OFC is damaged or underdeveloped, precise calculation of the value of specific action is difficult and an impulsive action is more likely to be chosen.

Another behavioral characteristic deeply associated with OFC damage is the difficulty of reversal learning (Schoenbaum et al., 2007). In reversal learning paradigm, when contingency of stimulus-reinforcement is altered, the new stimulus-reinforcement association is learned only if prior response is changed. However, a monkey with OFC lesion is not able to control responses from prior reinforcement and exhibits perseveration, although it cannot receive further reinforcement (Mishkin, 1964). This is due to the inability to update values of prior actions through negative feedback. Thus, the main function of the OFC is to calculate and update the value of an action through learning new stimulus-reinforcement association.

Unlike laboratory settings, reality poses many issues to consider when making decisions because the outcome is uncertain or risky in many cases. Therefore a precise representation of value judgment is thought to be quite advantageous in suppressing impulsive actions. Recent neuroimaging studies suggest that opportunity for choice is desirable (Leotti et al., 2010) and the anticipation of choice is also rewarding (Leotti and Delgado, 2011). For children or adolescents, however, whose prefrontal regions including the OFC are underdeveloped, the lack of experience limits the representation on values. Therefore, we need to frequently inform them on utility values of learning, provide opportunity to make their own choices, and enhance the quality of value judgment.

## Regulation of Motivation: Goal-Directed Control Process

How do people regulate their motivation? The reason people fail to perform tasks persistently and give in to temptation is because immediate rewards are highly favored over delayed rewards. Subjective values of rewards change with the point in time when the rewards are given and immediacy itself plays a relatively important role. If we vary the time and the magnitude of the reward, offer a number of options, and ask the participants to choose one, then they experience a conflict between small but immediate reward and large but delayed reward. One clear point is that as the reward is delayed, the relative value of the reward is decreased. This is called temporal discounting or delay discounting. Temporal discounting is directly related to self-control (Rachlin, 1995) or delay of gratification (Mischel and Gilligan, 1964), and is very similar to resisting temptation or suppressing impulse in its nature. Self-control and delay of gratification refer to the ability to select larger delayed over smaller immediate rewards.

McClure et al. (2004) conducted a study to search the brain areas associated with temporal discounting. They manipulated various monetary reward options at different times (e.g., $20 today vs. $25 after 2 weeks) and compared brain activation patterns during choice. The results showed that the striatum and the MOFC were activated when the immediate reward was selected, whereas the fronto-parietal cortex was activated when the delayed reward was selected. This indicates that selecting the immediate reward activates the reward and value pathway, but to delay immediate gratification, the prefrontal cortex responsible for cognitive control should be involved.

What makes people resist to temptation and control motivation to constantly pursue a specific goal? Controlling impulses and regulating motivation calls for a detailed planning and execution for future goals. The cognitive control is a central process underlying such regulation, including goal maintenance, planning, performance monitoring, strategy selection, and outcome evaluation. Therefore, the mechanism by which the impulse is controlled should not be understood as a mere suppression of desire, but it should be construed as a goal-directed regulation by a cognitive control.

Cognitive control is actually a very useful coping strategy to modulate motivation to deal with negative RPE or negative feedback. When it happens, the dopamine system becomes less activated decreasing the frequency of target action, ultimately eliminating the learned action. Emotional reactions to negative feedback do no good to control motivation. Rather, one should check for problems of the performance through cognitive control and modify strategies. This may lead to better performance and produce a positive RPE, which in turn stimulates dopamine release to promote motivation and raises the chance of a new learning.

Brain regions associated with cognitive control process are the ACC, the DLPFC, and the OFC (Cole and Schneider, 2007). The ACC which is responsible for executive functions involves in integration of cognition and emotion, attentional control, performance monitoring, error detection, response inhibition, planning of higher-level action, and strategy modification (Holroyd and Coles, 2002). The dorsal part of ACC, which is connected to the DLPFC responsible for working memory, is involved in cognitive functions. On the other hand, the ventral part of ACC is associated with emotional functions (Bush et al., 2000).

Social cognitive neuroscience studies on delay discounting found that individual variability in self-control has been due to the difference in the working memory capacity (e.g., Shamosh et al., 2008). From meta-analysis, Shamosh and Gray (2008) revealed a negative correlation (*r* = −0.23) between delay discounting and intelligence. Other studies also found that activation of the DLPFC has a strong correlation not only with the working memory capacity, executive function, and intelligence but with success rate of various delay of gratification tasks (Knoch and Fehr, 2007; Shamosh et al., 2008). Delay discounting tasks require carrying out cognitive and emotional control simultaneously while calculating the value of selected action. Thus, individuals who are capable of efficiently utilizing working memory have advantages. The DLPFC are recruited during the goal-directed behavior and top-down regulatory processes, including goal maintenance, strategic behavioral planning, and implementation of actions (e.g., Miller and Cohen, 2001; Tanji and Hoshi, 2008). Therefore, self-regulation can be regarded as the process of encoding the value of the goal into VMPFC to make goal-directed decisions and regulating them in the DLPFC (Hare et al., 2009).

A study on brain development in response to negative feedback conducted by Crone et al. (2008) demonstrated that similar activation pattern to that of adults were seen in the OFC for 8–11 age group, and in parietal cortex for 14–15 age group, but no activation was seen in the ACC and the DLPFC up until the age of 14–15. As the DLPFC and the ACC are the regions associated with cognitive control, no activation means that children and early adolescents pose difficulty in reflecting the behavior and searching for alternatives after receiving negative feedbacks. Because this finding suggests that the effort to change the behaviors of children through negative feedback might be ineffective, we need to develop an appropriate feedback system for children and adolescents.

Because self-control is an important cognitive ability that is linked to a wide variety of measures of academic achievement, it would be meaningful to develop self-control ability through training or intervention program. Fortunately, a growing number of studies have shown that working memory training improves cognitive control (Klingberg et al., 2002, 2005) as well as several other cognitive abilities, such as fluid intelligence and problem solving (Jaeggi et al., 2008). This suggests that improving cognitive controllability through working memory training is likely to be far more effective in promoting self-regulation rather than emphasizing the volitional power or boot camp-style training.

## An Integrative Perspective on Motivational Processes

**Figure 4:**
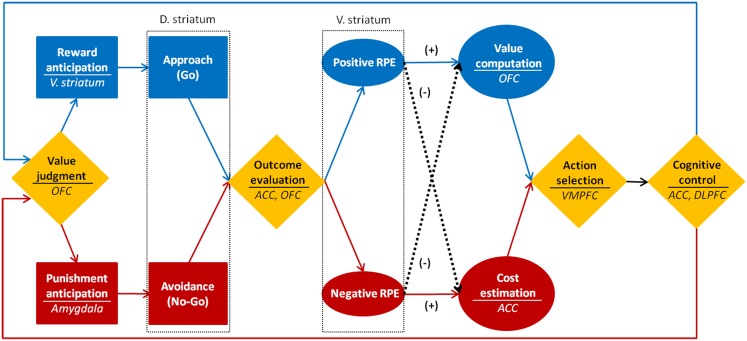
**Neuroscientific model of motivational process**.

By integrating neuroscientific findings on reward, learning, value, decision-making, and cognitive control, I propose a tentative model on motivational processes (see Figure 4). In the motivational process model, motivation is defined as a series of dynamic processes including generation, maintenance, and regulation of motivation of which primary functions are approach toward reward, learning through RPE, decision-making based on value, and cognitive control for goal pursuit. These sub processes interact with each other by sending prediction error signals from the striatum to the prefrontal cortex.

First, the motivation generation process is the process in which approach behavior is caused by the anticipation of reward. It is a process of either determining approach/avoidance behavior (Go/NoGo decision) or selecting an action among alternatives, based on the reward value. The reason for a motivation being generated for a previously unmotivated action is because the reward contingent upon a specific action increases the expectation for the reward, which continuously causes approach behavior. The generation of motivation can be judged by the frequency and duration of the approach behavior. The ventral striatum and the amygdala play a significant role in this process. Critical factors for the motivation generation process are incentive salience and reward anticipation from past experiences. And the enemies for this process are the punishment and high level of task difficulty.

Next, learning through positive RPE is a process to continuously maintain motivation. The stimulus-action-reward association is learned to raise the possibility of acquiring the subsequent rewards. When the reward is better than expected, the positive RPE occurs. The larger the error is, the bigger the learner’s surprise becomes. And the surprise leads to intense learning (Kamin, 1969; Rescorla and Wagner, 1972). Engraved stimulus-action-reward association enhances the value of a selected action and sustains the target behavior. The negative outcome evaluation, however, wears out positive RPE, which in turn reduces the effect of learning and the value of an action. In the motivation maintenance process, the positive RPE and value judgment are the crucial factors, and experiences of failure and perceived costs are the enemies. The sustaining of motivation can be measured by the frequency of the action selection, persistence, and efforts. The striatum and the OFC are the main brain regions involved in sustaining motivation.

Lastly, the cognitive control by the negative RPE is a process of self-regulation in fail-to-get-reward situation to pursue goal-directed behaviors and modify plans and strategies to explore new rewards. When the expected reward is not given as a result of an action, the value of the action decreases and the frustration grows bigger leading to diminished approach behaviors, and other alternative actions become massively attractive. This temptation can be resisted by successful cognitive control such as retrieving the long-term goal, monitoring the current performance, establishing a concrete plan, and selecting a new strategy. Motivations can also decline when rewards are always expected because the positive RPE stops increasing. Even in this circumstance, motivation can be promoted through the engagement of the goal-directed cognitive control process of establishing a new goal, plan, and strategy. The enemies for motivation regulation are the immediate impulse, the low executive processing capacity, and the lack of specific goals and plans. Delay of gratification and goal attainment are the barometers for motivation regulation. The ACC and the DLPFC are the main neural circuits related to the motivation regulation process.

A typical example of this motivational process can be easily found in an academic environment. Consider a situation where a student’s motivation to learn is generated, sustained, and regulated. A student with no initial motivation to learn a specific school subject may form strong intentions to study the subject for the first time in her life after being complimented or recognized by a teacher (reward-driven approach). Studying harder to get the teacher’s praise unexpectedly leads to a better grade (positive RPE). As she already learned, through the association, what actions to take to keep the good grade, she would put in continuous efforts and be able to maintain the motivation to learn to some extent (value-based decision-making). However, if the grade no longer improves, no more compliments are given by a new teacher (negative RPE), or she gets so used to the compliment that the value of the reward starts to drop (decrease of positive RPE), then she is likely to be in danger of falling for other tempting stimuli. At this moment, by retrieving her long-term goal, she can monitor her current state of performance, modify the plan, and search for alternative strategies (cognitive control). As a result, she can delay the immediate gratification and succeed in motivation regulation.

## Educational Implications

The neuroscientific model of motivational processes suggests several educational implications which can be used to enhance motivation to learn. For instance, reward is an essential driving force in the learning environment because approach behavior would not occur without reward. To motivate the unmotivated, the learning process should be rewarding and interesting. Rewards do not have to be tangible ones. Reward in the classroom can be any stimulus which has positive expected values, including positive feedback, praise, interesting activity, utility, relevance, social support, and relatedness. It is important to find out and make a list of appetitive stimuli including a variety of compliments, enjoyable activities, interesting materials, positive feedback, and diverse and novel learning context which can activate the reward circuit of children and adolescents. Since the repetition of the same compliment tends to reduce positive RPE, it is desirable to introduce various reward contingencies in an unexpected way in order to sustain the motivation.

To maintain motivation, the value a specific object and action must be high enough to lead to an action selection. Because the value is learned through trial and error, providing choices in autonomous learning environments would be beneficial for students to form and update their own value. This kind of choice practice may eventually develop the brain regions related to valuation and decision-making. In case the motivation decreases, the roles of attention and working memory cannot be more important. Thus, it would be necessary to develop the training program for these executive functions and to examine its effectiveness. Besides, creating a detailed goal hierarchy between proximal and distal goals and developing specific action plans will help students overcome the failure and temptation. Since the motivational process model proposed in this paper is only a provisional model, more research is required to verify its validity.

Neuroeducation or educational neuroscience is the interdisciplinary research field which builds connection between education and developmental, cognitive, emotional, or social neuroscience. It aims at developing curriculums, learning strategies, teaching methods, learning material, intervention programs to enhance diverse types of learning and ultimately providing optimal learning environments (Ansari et al., 2012; Kim, 2012). Since neuroeducation is a relatively new academic field, the establishment of systematic research paradigm along with intensive research is expected to largely contribute to actual educational settings. With accumulated research findings in the field of neuroeducation, a great deal of progress is being made on the learning and development of cognitive, emotional, and social skills. Nonetheless, research on motivation definitely needs more attention. Neuroeducational research on motivation has advantages for understanding implicit and dynamic aspects of motivational processes because observation and self-report reveal limitations. Choosing a research topic which holds strong ecological validity in educational settings becomes crucial. In particular, more attention should be paid to pragmatic research to enhance students’ motivation to learn. For instance, if we can understand the neural mechanisms underlying motivational phenomena such as interest, curiosity, decision-making, addiction, risk-taking, and self-regulation, we can develop a variety of interest-based learning and instruction, curiosity-inducing textbooks, non-threatening tests, and self-control training programs. The neurodevelopmental characteristics of children and adolescents should also be taken into account to optimize the motivation-related brain functions. The neuroeducational approach is also expected to contribute to resolve controversial issues in existing motivation theories, and to propose creative theories of motivation beyond traditional conventions.

## Conflict of Interest Statement

The authors declare that the research was conducted in the absence of any commercial or financial relationships that could be construed as a potential conflict of interest.
